# Quality of life in patients with endometrial carcinoma: A Longitudinal Study

**DOI:** 10.1002/nop2.927

**Published:** 2021-05-19

**Authors:** Jiebing Chen, Qiaojian Zou, Xuanmin Chen, Xiaochen Liu, Sha Ding, Yinglei Mo, Shuzhong Yao

**Affiliations:** ^1^ The First Affiliated Hospital of Sun Yat‐sen University Guangzhou China; ^2^ Zhongshan School of Medicine Sun Yat‐sen University Guangzhou China

**Keywords:** endometrial cancer, longitudinal study, quality of life

## Abstract

**Aim:**

To investigate the quality of life in patients with endometrial carcinoma and provide theoretical basis for nursing care.

**Design:**

In this study, 69 patients diagnosed with endometrial carcinoma from 2016–2018 were included in the cohort.

**Methods:**

Sixty‐nine patients from our hospital who underwent endometrial cancer surgeries were selected. The SF‐36 was used to investigate and analyse the patients’ quality of life in the first, second and third months after their operations. Questionnaires were administered to analyse the factors affecting postoperative quality of life.

**Results:**

Quality of life for the second and third months was obviously better than that for the first month after the operation (*p* < .05). Based on multivariate regression analysis, we found that patients with higher family income had better quality of life after surgery(*p* < .05). These results can provide some guidance for daily nursing work after endometrial cancer operation.

## 1 INTRODUCTION

Endometrial cancer (EC) is the sixth most common female tumour worldwide, and its incidence is increasing each year. It is currently the most common female pelvic malignant tumour in the United States now (Faria et al., 2019), and a retrospective study showed that the incidence rate of endometrial cancer in white Americans has reached 19/100,000 (Lortet‐Tieulent et al., 2018). According to data from the World Health Organization in 2018, nearly 380,000 women were diagnosed with endometrial cancer worldwide, resulting in approximately 90,000 deaths; thus, this type of cancer should receive more attention (Bray et al., 2018). Quality of life refers to people's feelings about their position inside their culture and value system, including the concept of health, which consists of the living environment, psychological health and social support (Panzini et al., 2017; Skevington, 2002). Quality of life can reflect the health status of people from multiple dimensions. To date, studies on the quality of life in patients with endometrial cancer have mostly been limited to cross‐sectional studies, and longitudinal studies in this field are extremely rare (Ferguson et al., 2018; Robertson et al., 2019; Salehi et al., 2018; Shisler et al., 2018). The 36‐item Short Form Health Survey (SF‐36) is a well‐known quantitative tool for evaluating quality of life(Ware & Sherbourne, 1992). It systematically assesses eight dimensions of psychological and physiological health and provides powerful evidence on the effect of a disease on quality of life.

In our study, we used the SF‐36 to dynamically assess the quality of life of patients with endometrial cancer at different times after their operations. Specifically, this study also used multivariate regression analysis and discovered the risk factors for low quality of life after surgery. Based on these findings, some targeted and individualized disposes can be applied in daily nursing work for different kinds of patients. This can help improve their quality of life after operations.

## 2 METHODS

### 2.1 Study design

From October 2016–December 2018, 69 patients suffering from endometrial cancer who were treated in the gynaecology department of a tertiary hospital in Guangzhou were selected. All the participants met the following inclusion criteria: (1) diagnosed with endometrial cancer by pathological results, (2) first diagnosed and performed staging operations in our hospital, (3) without a history of neoadjuvant chemotherapy and radiotherapy and (4) returned to the hospital regularly for follow‐up. In addition, the exclusion criteria were as follows: (1) patients with uncontrolled primary tumours or signs of recurrence, (2) patients who suffered from other types of malignant tumours simultaneously and (3) patients with a history of mental diseases and difficulty cooperating with the follow‐up. The study adhered to the principles of the Declaration of Helsinki. Research Ethics Committee approval was obtained for this study. The approval included the participant information sheet and informed consent form signed by all participants.

### 2.2 Data collection

Questionnaires were uniformly distributed by trained investigators uniformly and collected by one‐to‐one interviews. Investigators described our research to those who met the inclusion criteria and obtained their informed consent. All participants completed the SF‐36 the first, second and third months after the operation. Participants were excluded if they stopped chemotherapy without authorization or went to another hospital for treatment at any point of the study.

### 2.3 Investigation tools

The questionnaire for basic information included patient age, marital status, work status, educational level, disease stages and complications. The Chinese version of the SF‐36 was adopted. This scale includes 36 items across 8 dimensions, including physical function (PF), role physical (RP), bodily pain (BP), general health (GH), vitality (VT), social functioning (SF), role emotional (RE) and mental health (MH). Scores for each item are counted and converted into standardized scores by (original score–lowest possible score)/ possible score range × 100, and the standardized scores of the eight dimensions are summed to obtain the SF‐36 total score. The average score of the SF‐36 ranges from 0–100 points, where 0 points and 100 points represent the worst and best health results, respectively.

### 2.4 Statistical analysis

All statistical analyses were performed using Statistical Package for Social Sciences (SPSS) 23.0 software. One‐way ANOVA was adopted for comparing the means of three groups, and the Bonferroni test was used for post hoc comparison. Univariate analysis was used to identify factors associated with quality of life, and those factors with a *p*‐value of <.10 were included in multivariate regression analysis. A *p*‐value of <.05 was considered statistically significant.

## 3 RESULTS

In our study, 69 participants completed the SF‐36 at the first, second and third months after their operations. All data were divided into three groups depending on the time since the operation. In addition, personal information from participants was collected from the first questionnaire. The general information of 69 patients is shown in Table 1.

**TABLE 1:** Description for general conditions of selected patients

Item	Frequency (*N*)	Percentage (%)
Age (mean ± standard deviation)	53.9 ± 8.45	
Marital status		
Unmarried	1	1.5
Married	68	98.5
Fertility condition		
Have given birth	66	95.6
Have not given birth	3	4.4
Educational experience		
Under high school	37	53.6
High school or more	32	46.4
Menstrual condition		
Normal	10	14.5
Disorder	11	15.9
Menopause	48	69.6
Mood		
Stable	22	31.9
Instable	47	68.1
Physical discomfort^a^		
No	8	11.6
Yes	61	88.4
Payment		
Self‐paying	6	8.7
Health insurance or others	63	91.3
Monthly income		
<5,000 yuan	27	39.1
5,000–10,000 yuan	24	34.8
>10,000 yuan	18	26.1
Employed condition		
No	10	14.5
Yes	59	85.5
Chronic diseases		
Yes	27	39.1
No	42	60.9
Clinical stage		
I	37	53.6
II	15	21.7
III	14	20.3
IV	3	4.4

^a^ Including nausea, vomiting, choking sensation in chest, anhelation, palpitation, easy to tired, poor appetite and inattention. Data were analysed by SPSS.

For total SF‐36 scores, we found a statistically significant difference between the three groups. Specifically, the total scores were significantly higher in the second month and the third month than in the first month after the operation(*p* < .05), but no significant difference was found between the second and the third months (*p* > .05). (Table 2).

**TABLE 2:** Scores of each dimension and total scores after operation

Time after operation	PF	RP	BP	GH	VT	SF	RE	MH	Total scores (Mean ± *SD*)
First month	54.64 ± 25.99	35.51 ± 45.46	36.04 ± 23.92	37.87 ± 21.57	46.67 ± 18.26	44.38 ± 26.04	36.71 ± 43.96	50.26 ± 22.94	342.08 ± 126.15
Second month	68.91 ± 33.30	22.10 ± 36.52	50.19 ± 28.24	46.62 ± 22.31	56.74 ± 20.91	56.80 ± 24.67	37.68 ± 37.88	60.64 ± 23.37	396.23 ± 132.45
Third month	72.06 ± 20.83	32.72 ± 46.97	55.54 ± 31.56	44.71 ± 17.94	57.21 ± 19.91	61.40 ± 19.64	43.14 ± 37.79	60.84 ± 23.84	427.95 ± 125.74

Abbreviations: BP, Bodily pain; GH, General health; MH, Mental health; PF, Physical function; RE, Role emotional; RP, Role physical; SF, Social functioning; VT, Vitality.

Next, univariate analysis was used to identify factors that influenced the difference between the total scores of the third and first months after the operation. (Table 3) Those factors with a *p*‐value of<.05 were included in multivariate regression analysis, including educational experience, monthly income and physical discomfort. After excluding the confounding factor, we noticed that monthly income had a considerable impact on the quality of life after operation (both *p* < .05), while higher monthly income led to better postoperative quality of life. (Table 4).

**TABLE 3:** Univariate analysis of total scores between the first and third month after operation

Variable	Regression coefficient	*SE*	*t*	*p*
Age	1.580	2.543	0.620	.537
Educational experience (control group: under high school)	110.224	41.100	2.680	.009
Menstruation condition (control group: menopause)				
Normal	24.691	61.808	0.400	.691
Disorder	23.302	64.587	0.360	.719
Mood (control group: stable)	−59.386	45.970	−1.290	.201
Physical discomfort (control group: no)	131.775	64.567	2.040	.045
Payment (control group: self‐paying)	−56.673	75.308	−0.750	.454
Monthly income	68.865	25.977	2.650	.010
Employed condition (control group: no)	5.671	9.783	0.580	.564
Chronic diseases (control group: yes)	−11.144	44.009	−0.250	.801
Clinical staging (control group: I)	−24.251	23.676	−1.02	.309

**TABLE 4:** Multivariate regression analysis of influence factors for life quality after operation

Variable	Parameter estimation	*SE*	*F*	*p*
Educational experience (control group: under high school)	72.541	43.382	1.67	.099
Physical discomfort (control group: no)	78.756	65.273	1.21	.232
Monthly income	54.537	25.903	2.11	.039

## 4 DISCUSSION

As one of the most common gynecological cancers, a growing number of studies have focused on the quality of life in patients with endometrial carcinoma, especially in the perioperative period, although most of them were cross‐sectional studies. Similar to our study, Ferrandina (Ferrandina et al., 2014) and Zandbergen (Zandbergen et al., 2019) have reported quality of life in endometrial carcinoma patients, with a follow‐up of up to 24 months. Nevertheless, there are no reports on short‐term postoperative quality of life in endometrial carcinoma patients. Differ from previous long‐term studies, endometrial carcinoma patients in our study would return to hospital for repeated adjuvant treatment within a short period of time after surgery. During this period, the postoperative follow‐up by interacting and communicating with doctors and nurses for appropriate intervention and guidance was beneficial to patients. In this study, we found that the quality of life in endometrial carcinoma patients within the first month postoperatively was significantly lower than that in the second and third months, and the monthly income was positively correlated with their postoperative quality of life.

Nowadays, the treatment of endometrial cancer is developing. Staging operation followed by adjuvant treatments including but not limited to chemotherapy and radiotherapy have been widely applied (Amant et al., 2018; Lu & Broaddus, 2020). Within the first month postoperatively, endometrial cancer patients not only experienced physiological pain caused by the operation itself, but also the powerlessness owing to the inability to self‐care and unknown pathological results. In some situations, adjuvant treatments especially chemotherapy are performed right after operation, usually within 1 month (Lu & Broaddus, 2020). For most of endometrial cancer patients, the sequential burden of operation and chemotherapy in a short period of time can lead to a significant decrease in quality of life, including but not limited to pain, postoperative weakness, nausea, vomiting, fatigue, myelosuppression and alopecia caused by chemotherapeutic drugs (Lorusso et al., 2017; Shiozawa et al., 2013). As a result, health education and psychological counselling are highlighted for their importance to improve quality of life. Previously, studies have thoroughly demonstrated the importance of pre‐operative education (Kruzik, 2009; O'Brien et al., 2013). Besides, the management of physical and psychological problems during the immediate postoperative phase might improve satisfaction with the surgery experience, and decrease complications and length of hospital stay (Ramesh et al., 2017). Learn from previous experiences, disease‐related knowledge and comprehensive pre‐operative education should be propagated by nurses to help patients better prepare for postoperative challenges. Additionally, nurses can encourage them to communicate with other patients and build up their confidence by telling some success stories. Moreover, comfortable physical and psychological postoperative care need to be provided by nurses to help patients recover quickly. As previously described (Knols et al., 2005; Spence et al., 2010), nurses can also schedule postoperative rehabilitative treatment regimens according to individualized self‐care abilities and encourage patients to perform appropriate physical exercises according to their own status, which can not only prevent postoperative complications, but also increase their confidence during recovery. Furthermore, family members should be actively and seriously involved in. It is demonstrated that family support is of great significance to dissipate the anxiety of the patient and enhance the outcomes of postoperative rehabilitation (Cardoso‐Moreno & Tomás‐Aragones, 2017; Fang et al., 2017). Communicating with family members is imperatively given because a close relationship to the patient helps to enhance the optimism and confidence. Family members can encourage patients to accept the actual facts about their physical health, improve their psychological status and compliance to the treatment regimen (Fang et al., 2017). Support from family members can also satisfy their physical and mental needs, contribute to a quick postoperative recovery and improve their quality of life (Zhu et al., 2018). Eventually, patients would gradually embrace the pain caused by their condition and take the resolution to start their new life, which is conductive to an immense boost in their quality of life.

We also noticed that the monthly income of patients plays an important role in their postoperative quality of life. This finding is consistent with the results reported by earlier studies (Blackburn et al., 2019; Jansen van Rensburg et al., 2017; Yilmaz et al., 2017). Endometrial cancer is a financially draining disease with a long duration, and many patients incur a tremendous economic burden while overcoming this disease. Patients with better financial conditions tend to display a greater degree of self‐determination, such as using expensive drugs with fewer side effects or choosing appropriate treatments on their own, which may remarkably improve their quality of life. Conversely, patients with poor incomes may experience more toxicity and psychological pressure, seriously reducing their quality of life (Group, 2017). Besides, poverty forces people to focus on money rather than their health and makes them miss the ideal treatment time and increase treatment‐related costs. Consequently, we need to pay more attention to patients with lower incomes. Side effects and complications should be recognized and prevented earlier to avoid unnecessary expenses. Likewise, more psychological guidance should be provided during chemotherapy to solve concerns and prompt recovery.

Agreed to previous studies (Kim & Kim, 2017; Wang et al., 2018), educational experience is also a principal factor influencing quality of life in cancer patients. In routine clinical practice, establishing a good communication with patients enables them to gain more health knowledge of their conditions and effectively reduce the psychological burden, which can obviously improve their quality of life. The educational level is positively correlated with the understanding of the disease and an active psychological construction to face the cancer. While conducting postoperative health education, doctors and nurses should adequately assess the learning and acceptance ability of patients. Postoperative health education should be performed differently for patients with low educational levels through increasing the frequency to strengthen their memory, and carrying out innovative methods such as instant messaging services or online meetings to promote their recovery. Pamphlets using simple language and comprehensible images can also be used (Wilson, 2011). The role of family members and communities should be highlighted while providing these patients’ health education (Cardoso‐Moreno & Tomás‐Aragones, 2017). Support from families and friends can further deepen the understanding of health education and help them face the disease better, which can improve their quality of life.

Similarly, univariate analysis also revealed that physical discomfort may reduce patients’ quality of life. As mentioned before, patients undergoing chemotherapy may suffer from different kinds of physical discomfort such as nausea, vomiting, choking sensation in the chest, dyspnoea and palpitation, which significantly influence their quality of life (Del Pozzo‐Magaña et al., 2015; Lorusso et al., 2017; Shiozawa et al., 2013). These physical sensations can lead to pessimism, despair and even rejection to treatment. Even though the available treatment strategies for endometrial cancer keep improving and postoperative quality of life in patients undergoing laparoscopic resection is significantly higher than in those treated by laparotomy, the physical discomfort caused by treatment still greatly affects patients’ daily life (de Boer et al., 2016). These potential adverse reactions greatly affect patients’ enthusiasm for follow‐up treatment. Doctors and nurses should give patients more information about the potential side effects before treatment such as myelosuppression or alopecia following chemotherapy. Most importantly, great changes in personal appearance will increase patients’ psychological burden and affect their daily life, especially young women. Hence, it is necessary to provide patients with detailed explanations regarding the causes and solutions to hair loss after chemotherapy. Assisting patients to prepare headscarves or wigs may equally help them to eliminate the unpleasant feeling from alopecia, reduce their anxiety and help them make through the follow‐up chemotherapy treatments. Moreover, the creation of non‐nausea and vomiting wards may alleviate patients’ anxiety and relieve their discomfort. In order to distract patients and enable them to have some fun, nurses can also utilize some individual nursing methodologies such as music therapy (Bradt et al., 2016), relaxation therapy (Sun et al., 2017) and psychological guidance.

In daily clinical work, most nurses bear a heavy working pressure. The California government signed a legislation and stipulated that in oncology wards, one nurse should be responsible for no more than four patients at the same time (Coffman et al., 2002; Spetz, 2004). However, an observation study showed that in high‐level general hospitals of China, a nurse usually takes care of eight patients during the day and even twenty‐three patients at night on average (Shen et al., 2020). Busy daily work will inevitably lead to the lack of care for every patient. In our study, we systematically analysed the postoperative quality of life of endometrial cancer patients within a short period of time. This longitudinal study for the first time helps nurses fully understand different requirements of endometrial cancer patients in different postoperative periods, and instruct nurses to provide proper and targeted care for patients. It not only effectively improves the quality of nursing care and patient satisfaction, but also enhances patients’ satisfaction and quality of life by preventing adverse events. Our results provide clinical references for practical nursing care of patients with endometrial cancer or other types of cancer.

As could be expected, this study also has some limitations. First of all, all the data were obtained via a self‐report questionnaire, and measurement errors caused by subjective factors may exist. Second, only 69 participants were included in our study. Finally, this study's participants were all from China, which implies that the results should be extrapolated cautiously. In future research, more longitudinal studies focusing on the patients’ quality of life should be conducted and more evidence for clinical care should be made available.

## 5 CONCLUSION

In summary, doctors and nurses should pay more attention to patients’ quality of life in the first month after endometrial cancer operation, especially patients with lower income, lower educational level and physical discomfort. We can fully utilize psychological counselling and humanistic care, help patients fully understand the disease and coordinate to treatment positively, relax patients and increase their confidence again. In addition, the important role of family members should also be taken seriously.

## CONFLICT OF INTEREST

None declared.

## AUTHOR CONTRIBUTIONS

Yao Shuzhong and Mo Yinglei contributed to the division of labour in the research group. Chen Jiebin contributed to the design of project. Chen Jiebin, Liu Xiaochen and Chen Minxuan contributed to the writing of draft. Ding Sha contributed to recording the content of the meeting and making a questionnaire. Zou Qiaojian contributed to data statistics.

## ETHICAL APPROVAL

Research Ethics Committee approval for this study was obtained from Ethics Committee of the First Affiliated Hospital of Sun Yat‐sen University. The approval included the participant information sheet and informed consent form signed by all participants.

## Data Availability

The data that support the findings of this study are available on reasonable request from the corresponding author. The data are not publicly available due to patient privacy reasons.
